# Supplementary material for: Gut Microbiome Alteration in HIV/AIDS and the Role of Antiretroviral Therapy—A Scoping Review
Source: Microorganisms. 2024 Nov 1;12(11):2221. doi: 10.3390/microorganisms12112221 (PMC11596264; doi:10.3390/microorganisms12112221)
Supplement: Supplementary file 1 [file microorganisms-12-02221-s001.zip › microorganisms-3262047-Supplementary material 1.docx]

**PubMed 2024.09.03.**

| **Search** | **Query** | **Results** |
| --- | --- | --- |
| #3 | Search: **#1 AND #2** Sort by: **Publication Date** | 3,347 |
| #2 | Search: **("Gastrointestinal Microbiome"[Mesh]) OR (microbiome) OR (gut microbiome) OR (gut dysbiosis) OR (metagenomics) OR (“Metagenomics”[Mesh]) OR (microbiota)** Sort by: **Publication Date** | 198,805 |
| #1 | Search: **("HIV"[Mesh]) OR ("Acquired Immunodeficiency Syndrome"[Mesh]) OR (human immunodeficiency virus) OR (HIV) OR (opportunistic infections) OR (PLHIV) OR (“AIDS-Related Opportunistic Infections”[Mesh]) OR (AIDS) OR (acquired immunodeficiency syndrome)** Sort by: **Publication Date** | [599,064](https://pubmed.ncbi.nlm.nih.gov/?term=%28%28%28%28%28%22HIV%22%5BMesh%5D%29+OR+%28%22Acquired+Immunodeficiency+Syndrome%22%5BMesh%5D%29%29+OR+%28%28human+immunodeficiency+virus%29%29%29+OR+%28%28HIV%29%29%29+OR+%28%28AIDS%29%29%29+OR+%28%28acquired+immunodeficiency+syndrome%29%29&sort=pubdate&size=200) |

**Web of science 2024.09.03.**

| **Search** | **Query** | **Results** |
| --- | --- | --- |
| #1 | (ALL=(microbiome) OR ALL=(gut microbiome) OR ALL=(gut dysbiosis) OR ALL=(metagenomics)) AND (ALL=(opportunistic infections) OR ALL=(PLHIV) OR ALL=(HIV) OR ALL=(human immunodeficiency virus) OR ALL=(AIDS) OR ALL=(acquired immunodeficiency syndrome)) | 4,138 |

**Embase 2024.10.16.**

| **Search** | **Query** | **Results** |
| --- | --- | --- |
| #1 | ((microbiome) OR ('gut microbiome') OR ('gut dysbiosis') OR (metagenomics)) AND ((HIV) OR (PLHIV) OR ('opportunistic infection') OR ('human immunodeficiency virus') OR (AIDS) OR ('acquired immunodeficiency syndrome')) | 4,134 |
